# Investigating *Bacillus amyloliquefaciens* VFS2 for *Vicia faba*‐*fusarium* wilt biocontrol and plant growth promotion under osmotic stress

**DOI:** 10.1002/ps.70078

**Published:** 2025-07-24

**Authors:** Imen Haddoudi, Moncef Mrabet, Jordi Cabrefiga, Isabel Mora, Haythem Mhadhbi, Emilio Montesinos

**Affiliations:** ^1^ Laboratory of Legumes and Sustainable Agrosystems (L2AD) Centre of Biotechnology of Borj‐Cédria (CBBC) BP. 901 Hammam‐Lif Tunisia; ^2^ University of Tunis El Manar Faculty of Sciences Tunis Tunisia; ^3^ Laboratory of Plant Pathology Institute of Food and Agricultural Technology‐CIDSAV, University of Girona, Campus Montilivi Girona Spain

**Keywords:** antifungal activity, cyclic lipopeptides, Indole‐3‐acetic acid, siderophores, osmoadaptation, *bacillus*, biocontrol

## Abstract

**BACKGROUND:**

A dual assault from fungal infections and abiotic stress factors such as drought and salinity increasingly threatens fava bean production. These adverse conditions not only reduce crop yield and quality but also create a more conducive environment for fungal pathogens to thrive. In this study, we aim to investigate the potential of *Bacillus amyloliquefaciens* strain VFS2 for the biocontrol of *Vicia faba*‐*Fusarium* wilt under drought and salt stress.

**RESULTS:**

The *in vitro* growth and sporulation of *B. amyloliquefaciens* VFS2 increased under salt and drought conditions (NaCl 0‐200 mM and PEG6000 0‐20%) in comparison to the unstressed control. The antifungal activity of VFS2 was screened against a broad‐spectrum of *V. faba*‐phytopathogenic fungi from the genera *Fusarium*, *Boeremia*, *Rhizopus* and *Rutstroemia* on PDA amended with NaCl (up to 200 mM) or PEG6000 (up to 20%), and the inhibition of fungal growth ranged from 50% to 98%. The ability of the VFS2 to produce various cLPs isoforms (iturins, fengycins, surfactins) under osmotic stress was demonstrated. The strain VFS2 also produces more IAA (up to 2.5 fold) and siderophores (up to 3 fold) under osmotic stress than in unstressed conditions. In biocontrol assays, VFS2 was effective in reducing *V. faba* root‐rot caused by *F. equiseti* VFF16 in saline soils (1.8 ≃ 1.9 ms m^−1^) and (up to 100%) under drought conditions (50% of water holding capacity).

**CONCLUSION:**

Our findings indicate that *B. amyloliquefaciens* VFS2 has the ability to control *Fusarium*‐wilt and boost plant growth in extreme conditions. © 2025 The Author(s). *Pest Management Science* published by John Wiley & Sons Ltd on behalf of Society of Chemical Industry.

## Graphical abstract

The osmoadapted *B. amyloliquefaciens* VFS2 showed strong antifungal activities against *Vicia faba‐*pathogenic fungi and produced significant amounts of cLPs isoforms, indole‐3‐acetic acid, and siderophores under osmotic stress. These properties were associated with a *Vicia faba Fusarium*‐wilt disease biocontrol under saline and water‐deficient conditions. Consequently, *B. amyloliquefaciens* VFS2 is a promising biocontrol candidate of *Fusarium*‐wilt diseases amid the challenges of climate change.

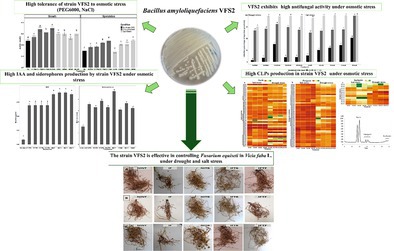

## 1 INTRODUCTION

Biological inoculants based on naturally occurring microbial antagonists provide an environment‐friendly alternative to control soil‐borne plant pathogens.^1^, ^2^ Several mechanisms of action have been described in antagonists, including direct competition for nutrients and niches, antibiosis, enzyme lysis, signal interference and induction of host resistance.^3^, ^4^, ^5^ Bacterial and fungal strains are the active ingredients of many commercial biopesticides.^6^ Despite their effectiveness under optimal conditions, it was reported that upon their introduction to the field, the biocontrol agents (BCA) survival is limited, and their population levels frequently decrease, resulting in a reduction of their effectiveness.^7^, ^8^ The shifts in microbial populations under field conditions are mainly attributed to the abiotic constraints having an adverse effect on the microorganisms and decreasing, therefore, their fitness in the plant environment.^9^ It was also recognized that the Plant Growth‐Promoting (PGP) activities are influenced by abiotic stress, and sensitive strains are not able to operate as plant growth promoters.^10^ Thus, stress tolerant microorganisms that live in close combination with the plant roots can ensure a significant improvement in plant growth and stress mitigation of cultivated crops under abiotic stress conditions.^9^, ^11^ Previous reports showed that the osmoadaptation of a BCA increased the biocontrol effectiveness of plant pathogens and was attributed to their capacity to produce endogenous osmolytes and to other complex physiological processes.^12^, ^13^ This was demonstrated for the BCAs *Pantoea agglomerans* EPS125, *Pseudomonas fluorescens* EPS288 and *Lactobacillus plantarum* PM411.^7^, ^12^, ^14^

*Bacillus* species have several characteristics that make them suitable for agricultural applications as good candidates for biocontrol agents of plant pathogens and to operate as plant growth‐promoting bacteria.^15^, ^16^, ^17^ *Bacillus amyloliquefaciens* and *B. velezensis* are components of plant and rhizosphere microbiota^15^, ^18^ and some strains among these species are commercially available as biocontrol agents.^18^, ^19^, ^20^, ^21^ *Bacillus amyloliquefaciens* harbors gene clusters that direct the synthesis of bioactive antifungal peptides (i.e., iturins, fengycins, surfactins, bacillomycin D) and polyketides (i.e., Baccillaenes, difficidins, macrolactins) by modularly organized mega‐enzymes like nonribosomalpeptide synthetases (NRPSs) and polyketide synthases (PKS).^16^, ^22^, ^23^ A substantial number of these metabolites have been described for their interest in agriculture, for their roles in suppressing disease in plants and for activating plant defence mechanisms against pathogens.^22^, ^24^ Despite these characteristics, less is known about the effect of salt and drought stress in the production of secondary metabolites by *B. amyloliquefaciens* and its effect on plant growth and biocontrol of fungal diseases.

Recently, we reported that among the rhizospheric bacteria associated with fava beans (FB), *B. amyloliquefaciens* strains were identified as the most abundant, producing a wide spectrum of secondary metabolites and exhibiting antifungal activity against FB infections fungi.^25^ Among the causal agents of *Fusarium*‐wilt disease in fava bean, *Fusarium equiseti* was found as the most prevalent fungus and strains assigned to this species showed a high tolerance capacity and pathogenic behavior under salt and drought conditions.^17^, ^26^ Therefore, the choice of BCA for field application in arid or semi‐arid conditions for fava bean should consider the bacterial fitness under extreme environments. In previous work, it was reported that *B. amyloliquefaciens* VFS2 is efficient in controlling *Fusarium*‐wilt disease of fava bean.^5^ In this context, the present study was designed to evaluate the effect of salt and drought stress on (1) the growth and sporulation of *B. amyloliquefaciens* VFS2, (2) the antifungal activity against fava bean‐pathogenic fungi, (3) the secondary metabolites production, (4) and the biocontrol efficacy of *Fusarium*‐wilt in FB.

## 2 MATERIALS AND METHODS

### 2.1 Growth conditions of *B. amyloliquefaciens* VFS2

The *B. amyloliquefaciens* VFS2 (GenBank accession no MK227454) was preselected among a collection of *Bacillus* spp. strains associated with *V. faba* plants on the basis of a broad‐spectrum of bioactive metabolites production and their high antifungal activity against most of the *V. faba‐*phytopathogenic fungi.^17^ VFS2 was maintained on Luria‐Bertani (LB) medium at 28 °C and stored at −80 °C in glycerol (25%). For the bioassays, VFS2 was cultivated on Production Medium (PM)^27^ amended with NaCl (0, 50, 100, 150, and 200 mM) for the salt stress and with different polyethylene glycol PEG6000 (0, 9, 15, 18, and 20%) for the drought stress.

The concentrations considered for NaCl and PEG6000 used in this work give the same osmotic pressure values on the medium; 183.18 10^−3^ MPa (50 mM and 9%), 446.34 10^−3^ MPa (100 mM and 15%), 652.74 10^−3^ MPa (150 mM and 18%), 864.3 10^−3^ MPa (200 mM and 20%), according to Chaouachi.^28^ Then, the culture media were incubated in a shaking incubator at 28 °C, 150 rpm at different times of incubation (0, 8, 24, 48, and 72 h), and three replicates for each treatment were done.

### 2.2 Fungal strains and growth conditions

Fungal strains used in this study are *F. equiseti* VFF16 (GenBank accession no. MK138378), *F. oxysporum* KLR14 (no. MK615110), *F. brachygibbosum* VFF2 (no. MK138381), *F. graminearum* VFF6 (no. MK141017), *F. equiseti* VFF12 (no. MK138372), *Rutstroemiaceae sp*. VFF7 (no. MK141018), *Rhizopus oryzae* VFF1 (no. MK138370) and *Boeremia exigua* VFF4 (no. MK138369). Those fungi were selected among a fungal‐strain collection previously isolated and identified on field‐grown fava bean plants.^17^ The strains are characterized by their high pathogenicity on *V. faba*, as well as their tolerance and aggressiveness under salt and drought stress conditions.^17^ Among the collection, *F. equiseti* VFF16 exhibited the highest virulence under abiotic stress conditions, making it the most suitable candidate for evaluating the robustness of biocontrol by VFS2. The fungal strains were maintained on potato dextrose agar (PDA) medium by regular sub‐culturing.

### 2.3 Effect of NaCl and PEG6000 on *B. amyloliquefaciens* growth and sporulation

The strain *B. amyloliquefaciens* VFS2 was grown on a PM medium amended with different concentrations of NaCl (0, 50, 100, 150, and 200 mM) and PEG6000 (0, 9, 15, 18, and 20%), and incubation times (0, 8, 24, 48, and 72 h), and the growth, sporulation and pH were measured. Decimal dilution series (10^−1^–10^−5^) were performed from each aliquot to determine cultured cells' number of VFS2 by dropping 20 μL of each tube on LB agar. The VFS2 spores' aliquots were heat activated for 10 min at 80 °C, and dilution series were performed. Colony‐forming units were counted after 24 h of incubation at 28 °C for each culture condition.

The area under the growth curve (AUGC) and the area under the sporulation curve (AUSC) were used to calculate the effect of salt and drought stress. Both areas under growth curves (AUGC and AUSC) were calculated according to the international model for the area under the growth equation.^29^

### 2.4 Effect of salt and drought stress on antifungal activity

#### 2.4.1 Antifungal activity on solid medium

To evaluate the antifungal activity of *B. amyloliquefaciens* VFS2 under salt and drought stress, the PDA medium was amended with different NaCl and PEG6000 concentrations, as described above. The antifungal activity of VFS2 was tested against *F. oxysporum* KLR14, *F. brachygibbosum* VFF2, *F. graminearum* VFF6, *F. equiseti* VFF12, *F. equiseti* VFF16, *Rutstroemiaceae sp*. VFF7, *R. oryzae* VFF1, and *B. exigua* VFF4.

A dual culture method on PDA medium according to Zhang *et al*.^30^ was used. Briefly, a fungal disc from pure culture (7 mm in diameter) previously grown on PDA medium was deposited in the center of the plate and VFS2 strain was streaked on the medium 20 mm from the dish edge, in triplicate for each concentration and incubated at 25 °C for 5–8 days. The plates containing only fungus for each concentration of NaCl and PEG6000 were used as controls. In order to assess the antifungal activity of the VFS2, a percentage inhibition was computed compared to a control.

#### 2.4.2 Antifungal activity on liquid medium

The strain VFS2 was cultivated on PM medium amended with different concentrations of NaCl (0, 50, 100, 150, and 200 mM) and PEG6000 (0, 9, 15, 18, and 20%) and incubated at a shaking incubator at 28 °C/150 rpm. Supernatants were collected separately at different incubation times (0, 8, 24, 48, and 72 h) and filtered later through 0.2 μm filters before use.

The *F. equiseti* VFF16 was used as the target fungus. In the microplate wells, the following components were mixed: 160 μL of PDB broth, 20 μL of VFF16 spores' suspension (10^6^ spores/mL), and 20 μL of VFS2‐cell free supernatant. 20 μL of PM medium amended with different concentrations of NaCl and PEG6000 was used as a non‐treated control. Three replicates for each treatment were made. The ELISA plates were incubated in the microplate reader (Varioskan) for 3 days at 25 °C under shaking with an automatic reading of optical density at 620 nm hourly during the 3 days of incubation.

### 2.5 Effect of salt and drought stress on cLPs production

*Bacillus amyloliquefaciens* VFS2 produces three types of cLPs: iturins, fengycins and surfactins.^25^ In order to assess the effect of salt and drought stress on the cLPs production, the VFS2 was cultivated on the PM broth medium amended with different concentrations of NaCl (0, 50, 100, 150, and 200 mM) and PEG6000 (0, 9, 15, 18, and 20%) and incubated in an orbital shaker (150 rpm) at 28 °C. cLPs were then extracted, purified and detected at 24, 48, and 74 h by HPLC according to Haddoudi *et al*.^17^ Different HPLC profiles were obtained depending on the growth conditions of VSF2 and compared, and the effect of stress on the production of cLPs was estimated after measuring the peak intensity for each profile than calculated using the following equation: $\mathrm{Ln}=\frac{\mathrm{Tr}-C}{C}$ where, Tr is the HPLC peak intensity of isoform under stress and C is the HPLC peak intensity in an unstressed condition.

### 2.6 Effect of salt and drought stress on IAA production

Indole‐3‐acetic acid (IAA) production under salt and drought stress was determined using the following medium: Sucrose 10 g·L^−1^, (NH_4_)_2_ SO_4_ 1 g·L^−1^, MgSO_4_ 7H_2_O 0.5 g·L^−1^, yeast extract 0.5 g·L^−1^, CaCO_3_ 0.5 g·L^−1^, NaCl 0.1 g·L^−1^, K_2_HPO_4_ 2 g·L^−1^ and L‐tryptophan 0.5 g·L^−1^, amended, separately, with different concentrations of NaCl (0, 50, 100, 150, and 200 mM) and PEG6000 (9, 15, 18, and 20%). Each treatment was triplicated. A 100 μL of overnight culture of strain VFS2 at 28 °C was transferred into 5 mL of the prepared media in 10 mL bottles and incubated at 28 °C for 5 d with agitation at 150 rpm. The cultures were subsequently centrifuged at 10 000 rpm for 5 min, and then 1 mL of supernatant was mixed with 2 mL of Salkowski reagent (12 g of FeCl_3_ per liter of 8.9 M H_2_SO_4_) and 100 μL of phosphoric acid (10 mM). Following incubation of the samples for 30 min at 25 °C, the optical density was measured at 520 nm. The appearance of pink color indicates the production of IAA. To calculate the concentration of IAA for each treatment, a standard curve ranging from 0 to 100 μg ml^−1^ of pure IAA (Indole‐3‐acetic acid, MP Biomedicals, inc.) was used.

### 2.7 Effect of salt and drought stress on siderophores production

Siderophores production under salt and drought stress was determined using Chrome Azurol Sulfonate (CAS) agar medium^31^ amended with different concentrations of NaCl (0, 50, 100, 150, and 200 mM) and PEG6000 (9, 15, 18, and 20%). Each treatment was triplicated. The siderophores production was measured according to the protocol of Shin *et al*.^32^ The appearance of orange color on the CAS agar plates around the wells indicates the production of siderophores. Finally, the diameters of the haloes were measured.

### 2.8 Biocontrol of *F. equiseti* VFF16 infection in FB plants under salt and drought stress

The biocontrol assay was performed with potted fava bean plants grown in soil samples. The soil used in this study was collected from the Soliman region (North‐East of Tunisia) which belongs to the semi‐arid bioclimatic area. Soliman region is an agricultural zone close to a lake, and two types of soil were taken: saline and non‐saline. Soil salinity during the sampling was determined with a field probe (Spectrum, Technologies, Inc. Item #2265FST24P, Field Scout Direct Soil 24 T‐Handle EC Probe). The soils sampled were non‐saline (at GPS zone: 36.704937; 10.431889: T17 °C, salinity 0.342 ms·m^−1^), and saline (at GPS zone: 36.705053; 10.431570; T18 °C, salinity 1.8 ‐ 1.9 ms·m^−1^).

The soils were three times autoclaved at 120 °C for 20 min on three consecutive days, in order to eliminate soil biota^33^ and transferred into cleaned polyvinyl chloride pots of 5 kg. The pot's weight was adjusted and closed with lids before starting the biocontrol assay.

The biocontrol experiment consisted of five treatments, where we adopted the terminology ‘treated’ for the bacterium, and ‘inoculated’ for the fungus; NINT: non‐inoculated and non‐treated; IF: non‐treated and inoculated with *F. equiseti* VFF16; NITB: Treated only with *B. amyloliquefaciens* VFS2; IFTB: inoculated with *F. equiseti* VFF16 and treated with strain VFS2; IFTF: inoculated with *F. equiseti* VFF16 and treated with the fungicide Benomyl (0.5 g/L). All the treatments were carried out under three systems: unstressed condition, salt stress using saline soil, and drought stress with reduction of irrigation water to 50% of water holding capacity. Five plant replicates for each treatment under each condition were considered.

The inoculum of *F. equiseti* VFF16 was prepared using sterilized millet seeds (autoclaved at 120 °C for 20 min) according to the protocol of Bahroun *et al*.^34^ VFF16 was grown in a 1 L flask containing 100 g millet seeds and 100 mL sterile distilled water at 25 °C in darkness for 3 weeks. Then, the fungal inoculum was filtered through cheesecloth, and sterile distilled water was used to wash the millet seeds. The VFF16 spores' suspension was used to infest the soil samples by mixing the sterile soils with 10^6^ spores/g soil for treatments involving fungal infection, and infested soil samples were incubated for 1 week (darkness at 25 °C in the greenhouse/Photoperiod (16 h/8 h)/25 °C). In the meantime, seedlings of *V. faba* Minor var. Bachar (Tunisian seeds cultivar) was prepared as described in Haddoudi *et al*.^35^ While *B. amyloliquefaciens* VFS2 was grown on PM broth for 3 d and adjusted to 10^6^ CFU/mL as the bacterial inoculum. One week after the soil incubation, the seedlings were dipped in roots incubated for 1 h in *B. amyloliquefaciens* VFS2 suspension and later transplanted into pots. After the transfer, 50 mL of VFS2 suspension was applied to the soil around the roots of the seedlings for the biological control treatment. A chemical‐fungicide control treatment (inoculated with *F. equiseti* VFF16 and treated with chemical fungicide) was included by pouring 50 mL of benomyl solution (0.5 g/L) on the soil surface around the plant.

Plants were maintained in the greenhouse under controlled conditions and irrigated with sterile distilled water. Under drought stress, plants were irrigated with 50% of the soil‐water holding capacity. The effect of the treatments was determined after 60 days using the growth and disease parameters; root‐system growth was photographed, and root‐rot was checked. Root Dry Weight (RDW) and Shoot Dry Weight (SDW) were measured according to Haddoudi *et al*.^25^

### 2.9 Statistical analysis

The analysis was performed with the R software version 4.2.2 (R Core Team, 2023) for various recorded‐parameters (ANOVA, means comparisons) and graphs.^36^ Multiple mean comparison was performed using the Tukey test (pairwise comparisons) at *P* = 0.05. The heatmaps and hierarchical clustering of cLPs production were performed using the MetaboAnalyst version 4.0 software.

## 3 RESULTS

### 3.1 Growth and sporulation under stress conditions

**Figure 1:**
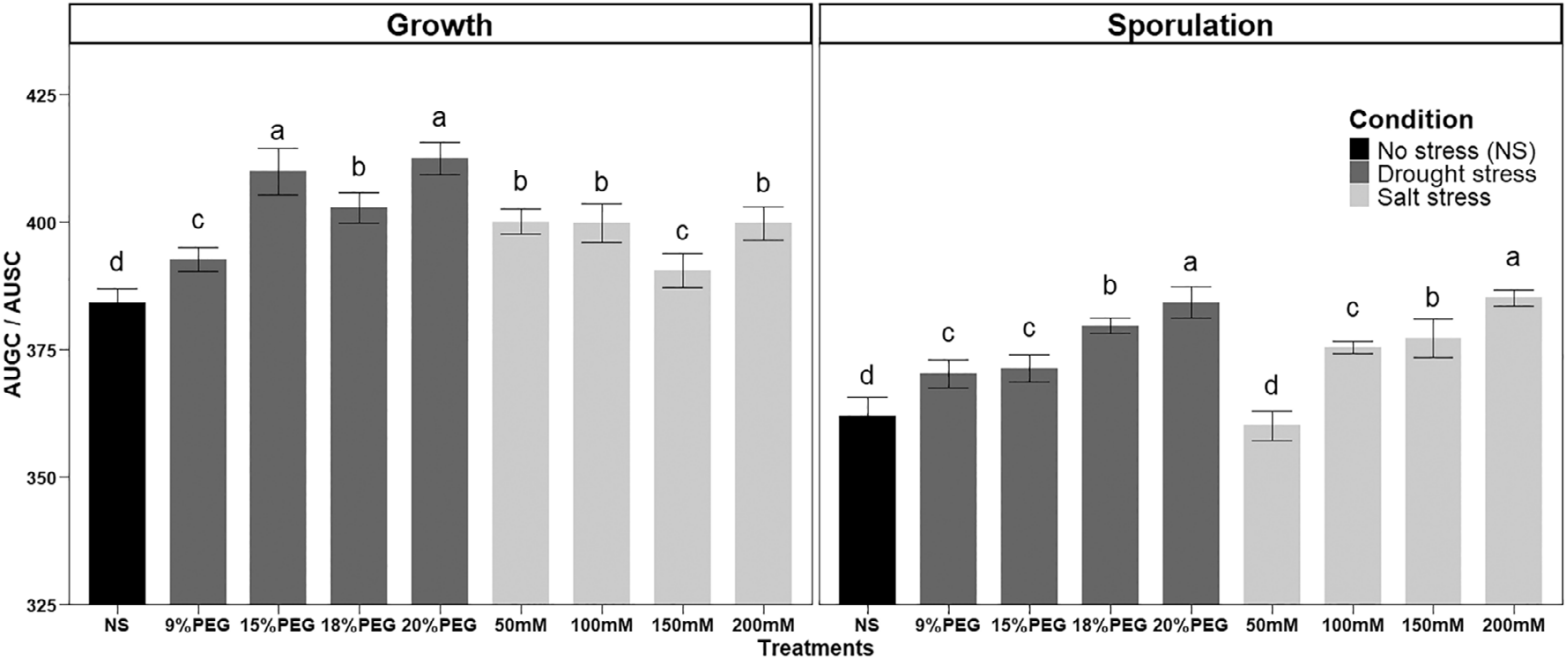
Growth and sporulation of *B. amyloliquefaciens* VFS2 under salt and drought stress. The experiment was performed on PM medium amended with NaCl (0, 50, 100, 150, and 200 mM) and PEG6000 (0, 9, 15, 18, and 20%). The area under the growth curve (AUGC) and area under the sporulation curve (AUSC) were calculated after 0, 8, 24, 48 and 72 h of incubation. Histograms with different letters are statistically different (*P* < 0.05, Tukey test). Bars represent the standard deviation between replicates for each treatment.

The effect of salt and drought stress on the growth, sporulation and pH of the growth medium of *B. amyloliquefaciens* VFS2 was analyzed on PM medium amended with different concentrations of NaCl and PEG6000. During the fermentation phase, the pH was stable under both stresses (pH 6) (Data not shown). However, under various NaCl and PEG6000 concentrations, the growth and sporulation of the *B. amyloliquefaciens* VFS2 significantly increased (*P* < 0.001) compared to data of unstressed control (Fig. 1). The growth of VFS2 strain was significantly higher (*P* < 0.001) under drought stress when comparing PEG6000 *versus* NaCl concentrations having the same osmotic pressure (9% *vs*. 50 mM, 15% *vs*. 100 mM, 18% *vs*. 150 mM, and 20% *vs*. 200 mM) (Fig. 1). However, the sporulation of VFS2 under stress was significantly higher than value obtained under unstressed condition (*P* < 0.001) but is of the same magnitude between drought and salt stress (*P* < 0.001).

### 3.2 Effect of salt and drought stress on the antifungal activity

#### 3.2.1 On solid medium

**Figure 2:**
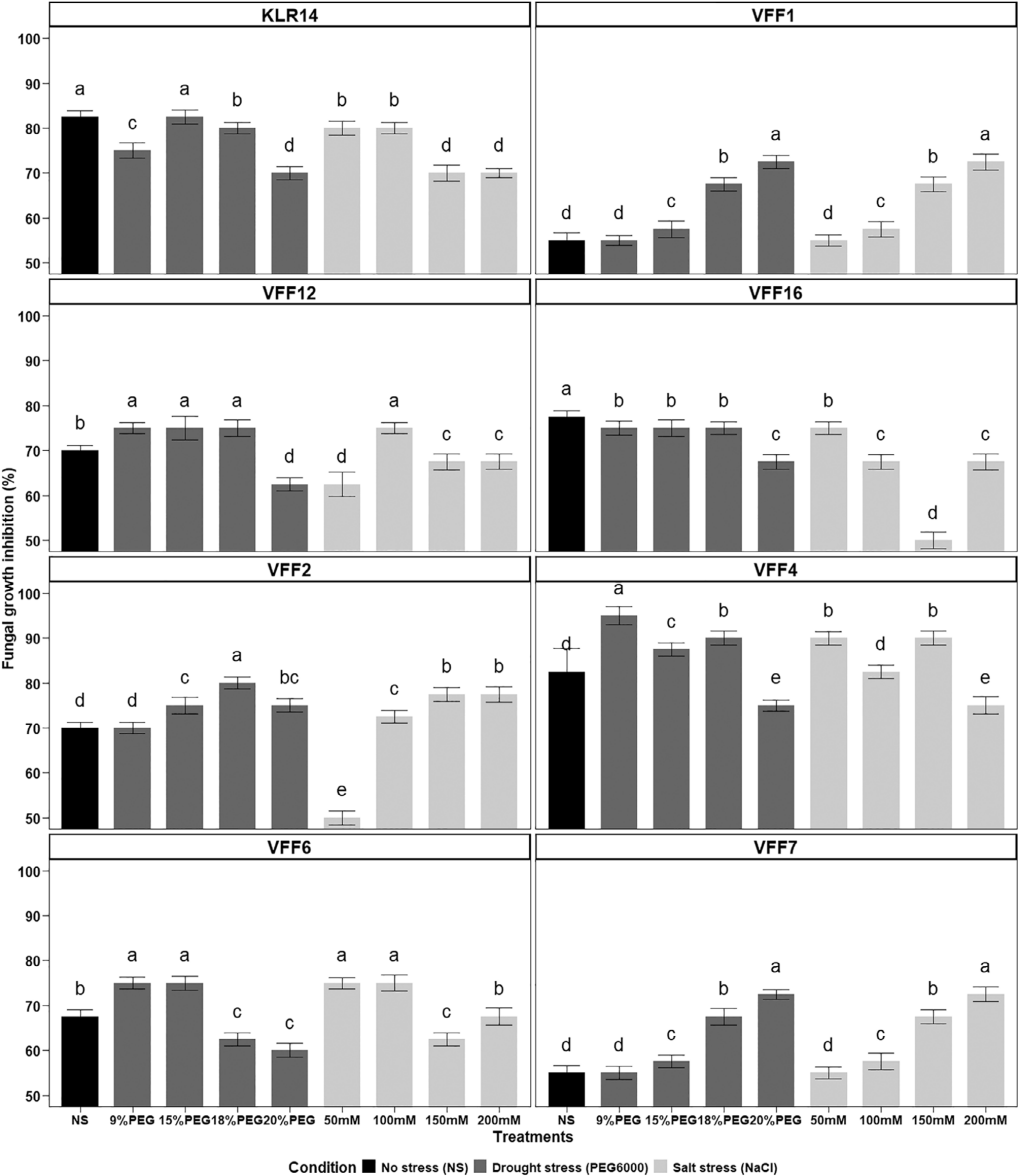
Growth inhibition of the phytopathogenic fungi *F. oxysporum* KLR14 (a), *R. oryzae* VFF1(b), *F. equiseti* VFF12 (c), *F. equiseti* VFF16 (d), *F. brachygibbosum* VFF2 (e), *B. exigua* VFF4 (f), *F. graminearum* VFF6 (g), and *Rutstroemiaceae sp*. VFF7 (h) by *B. amyloliquefaciens* VFS2 grown at different salt and drought stress conditions. The experiment was performed on PDA medium amended with different concentrations of NaCl (0, 50, 100, 150, and 200 mM) and PEG6000 (0, 9, 15, 18 and 20%). Histograms with different letters are statistically different (*P* < 0.05, Tukey test). Bars represent the standard deviation between replicates for each treatment.

The antifungal activity of *B. amyloliquefaciens* VFS2 was checked against eight pathogenic fungal strains on a PDA medium amended with different concentrations of NaCl and PEG6000. Under control treatment (non‐amended medium), VFS2 inhibited all tested fungal strains, and the inhibition percentage ranged from 55% (case of *R. oryzae* VFF1) to 87.5% (case of *Rutstremiaceae sp*. VFF7). When the growth medium was amended with NaCl (up to 200 mM) and PEG6000 (up to 20%), the strain VFS2 maintained the ability to inhibit the mycelium growth of the tested phytopathogenic fungi (Fig. S1). The percentages of inhibition were, in most cases, statistically similar or higher than values obtained under non‐treated control conditions, particularly at 50, 100, and 150 mM NaCl and 9, 15, and 18% PEG6000 (Fig. 2). Compared to the control of each fungus, the increases in inhibition were noticed for *B. exigua* VFF4, *F. brachygibbosum* VFF2, *F. equiseti* VFF12, VFF16, and *R. oryzae* VFF1, and were up to 90% at 150 mM NaCl and 18% PEG6000 for *B. exigua* VFF4. However, a decrease in inhibition was noticed for *Rutstremiaceae sp*. VFF7 when increasing NaCl and PEG6000 concentrations in the growth medium, from 87.5% under control conditions to 62.5% under 150 mM NaCl and 9% PEG6000. Overall, *B. amyloliquefaciens* VFS2 was able to inhibit all tested fungal strains' growth by more than 50% at various NaCl and PEG6000 concentrations.

#### 3.2.2 On liquid medium

**Figure 3:**
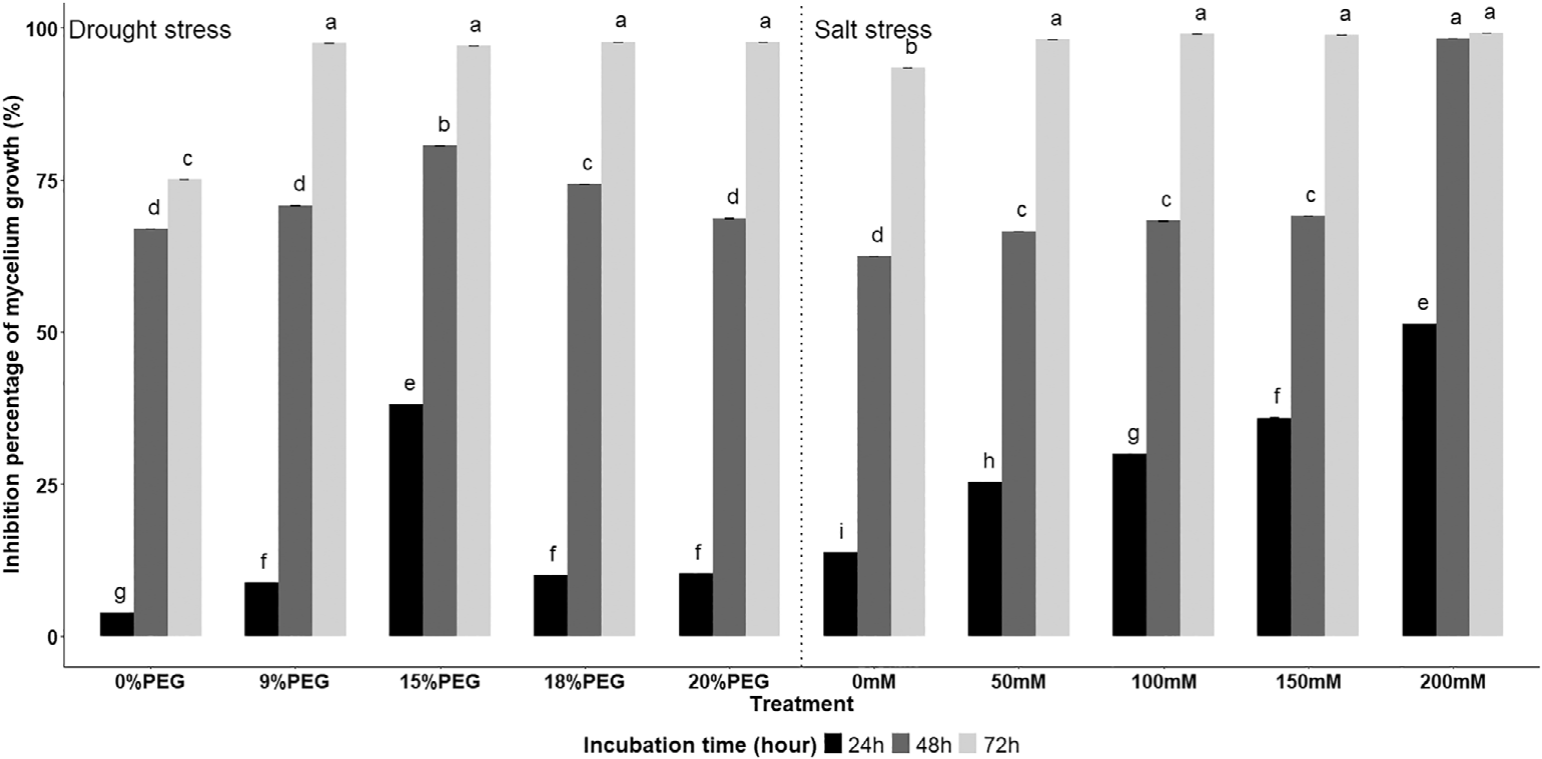
Antifungal activity of cell‐free supernatant of *B. amyloliquefaciens* VFS2 against *F. equiseti* in ELISA microplate. Optical density of the fungal spores' suspensions is measured at 620 nm. The treatments consisted of using VFS2 supernatant‐previously cultivated on PM medium amended with NaCl (0, 50, 100, 150, and 200 mM) (a) and PEG6000 (0, 9, 15, 18, and 20%) (b). The spores' suspensions and VFS2 supernatant were incubated at 24, 48, and 72 h. Bars represent the standard deviation of three independent replicates. PDB, potato‐dextrose broth medium.

Antifungal activity of *B. amyloliquefaciens* VFS2 against *F. equiseti* in liquid medium was significantly influenced by abiotic stress conditions (*P* < 0.0001), as revealed by OD₆₂₀ readings (Fig. 3). Under both abiotic stresses, inhibition of fungal growth generally increased with higher stress concentrations and longer incubation periods of strain VFS2.

Under salt stress, an increase in inhibition of fungal growth was noticed with the increase of NaCl concentration. The inhibition varied from 31 to 55% at 24 h and from 14 to 87% at 48 h under 50 mM and 200 mM NaCl, respectively. It was more noticeable after 72 h of *B*. *amyloliquefaciens* VFS2 incubation reaching values of 98 and 99% at 50, 100, 150, and 200 mM NaCl (Fig. 3(a)). Under drought stress, similarly, the inhibition of fungal growth increased in the presence of PEG6000 concentration compared to the control and time of incubation. It ranged from 15 to 44% at 24 h of incubation, from 20 to 56% at 48 h, and from 94 to 98% at 72 h, at 9% to 15% PEG6000 (Fig. 3(b)). Notably, 15% PEG resulted in the highest inhibition at 24 and 48 h, compared to other concentrations. However, after 72 h, antifungal activity remained consistently high and comparable across all PEG concentrations. These findings indicate that while *B. amyloliquefaciens* VFS2 exhibits some variability in early responses to stress, it achieves robust and consistent antifungal activity at later incubation times.

### 3.3 Effect of salt and drought stress on cLPs production by *B. amyloliquefaciens* VFS2

The effect of salt and drought stress on the production of iturins, fengycins and surfactins was measured by HPLC at 24, 48, and 72 h of incubation under different NaCl and PEG6000 concentrations. Cultures supernatants from 0 and 8 h incubation were eliminated because there was no antifungal activity.

**Figure 4:**
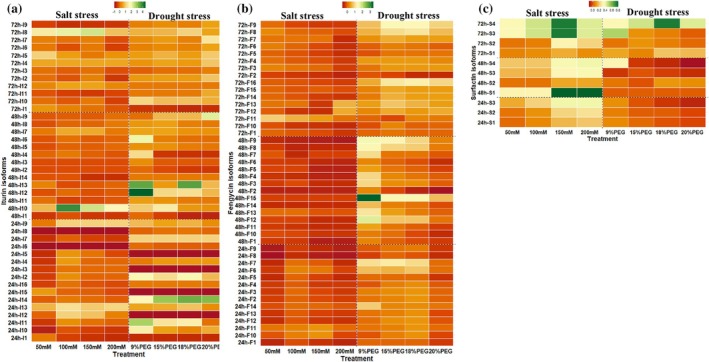
Heatmaps of iturins (a), fengycins (b) and surfactins (c) produced by *B. amyloliquefaciens* VFS2 on PM medium amended with NaCl (0, 50, 100, 150, and 200 mM) and PEG6000 (0, 9, 15, 18, and 20%) and at different incubation times (24, 48, and 72 h). Results were computed using R studio. The color scale indicates the level of isoform production under salt and drought stress compared to conditions without stress.

Heatmap analysis and hierarchical clustering showed a relationship between each isoform with the different concentrations of NaCl and PEG6000 (Fig. 4). Cluster analyses showed that the isoforms of iturins (Fig. 4(a)), fengycins (Fig. 4(b)) and surfactins (Fig. 4(c)) were segregated into groups according to their corresponding production profiles under different concentrations of NaCl and PEG6000 and incubation times. The hierarchical clustering on the left side and downside of the heatmaps for iturins, fengycins and surfactins showed that the various isoforms of each CLP were differentially produced for the same treatment of NaCl and PEG6000, as well as between NaCl and PEG6000 treatments.

Interestingly, the production of the major fraction of iturin and fengycin isoforms is maintained under various NaCl and PEG6000 concentrations, which may explain the high antifungal activity of the strain VFS2 under salt and drought stress against different phytopathogenic fungi.

### 3.4 Effect of salt and drought stress on IAA and siderophores production by *B. amyloliquefaciens* VFS2

The amount of IAA production in the tryptophan medium increased significantly (*P* < 0.0001) due to the different concentrations of NaCl and PEG6000 compared to the unstressed medium (Table 1). There was also a significant difference (*P* < 0.0001) between the two types of stresses, where the strain VFS2 produced more IAA under salt stress than under drought stress (Table 1). Compared to the control treatment, VFS2 produced 2 folds and 2.5 folds more IAA in medium amended with PEG6000 (9–20%) and with NaCl (50–200 mM), respectively.

**Table 1:** Indole‐3‐acetic acid (IAA) and siderophores production in *B. amyloliquefaciens* VFS2 under salt and drought stress

Treatment	Concentration (%PEG, mM NaCl)	IAA (μg mL^−1^ ± SD)	Siderophores (Halo mm ± SD)
Control	0	29.5 ± 0.9^c^	1.5 ± 0.5^d^
PEG6000	9	56.7 ± 1.1^b^	3.5 ± 0.3^c^
	15	57.5 ± 1.1^b^	3.2 ± 0.1^c^
	18	57.5 ± 1.1^b^	3.8 ± 0.1^b^
	20	57.5 ± 1.1^b^	3.2 ± 0.1^c^
NaCl	50	72.7 ± 2.3^a^	4.7 ± 0.2^a^
	100	75.1 ± 2^a^	3.6 ± 0.0^c^
	150	75.1 ± 1.8^a^	3.7 ± 0.0^b^
	200	72.7 ± 1.8^a^	3.2 ± 0.2^c^

*Note*: The growth medium is amended with NaCl (0, 50, 100, 150, and 200 mM) for salt stress and with PEG6000 (0, 9, 15, 18, and 20%) for drought stress. Each production value is the average of three replicates [±standard deviation (SD)]. In the same column, values with different letters indicate significant differences according to the Tukey test (*P* < 0.05).

The production of siderophores in VFS2 increased significantly (*P* = 0.04) under PEG6000 (9–20%) and NaCl (50–200 mM), compared to their production in a non‐amended medium (Table 1). The increases were 2 and 2.3 folds at 20 and 18% PEG6000, respectively. Under salt stress, the production of the siderophore increased by 2.8 and 1.9 folds at 50 and 100 mM NaCl, respectively, where there was no significant difference between drought and salt stress (*P* = 0.69).

### 3.5 Biocontrol of *fusarium‐*wilt disease in fava bean using *B. amyloliquefaciens* VFS2 under stress conditions

The biocontrol efficacy of *B. amyloliquefaciens* VFS2 against *F. equiseti* VFF16 in *V. faba* was assessed in autoclaved‐soil samples under three growing systems, including unstressed, salt‐stressed and drought‐stressed conditions. The inoculation of *F. equiseti* VFF16 reduced the root‐systems growth of *V. faba* plants under the three growing systems (Fig. S2). However, the inoculation of *B. amyloliquefaciens* VFS2 to infected plants with the *F. equiseti* VFF16 has re‐established the root‐systems growth, similar to the control (NTNI) and IFTF plants (plants infected with VFF16 and treated with Fungicide). Strain VFS2 reduced *V. faba* root‐rot induced by *F. equiseti* VFF16 under salt and drought stress in the same order as under unstressed conditions (Fig. S2).

**Figure 5:**
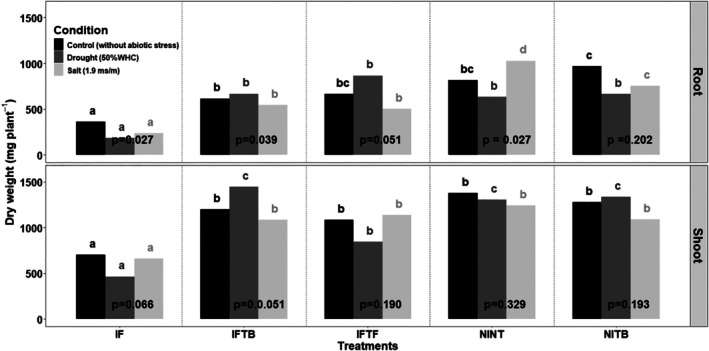
Histogram of dry weight of root (RDW) and shoot (SDW) of *V. faba* plants in the biocontrol assay of *F. equiseti* VFF16 using the *B. amyloliquefaciens* VFS2. The treatments were IF, inoculated with *F. equiseti* VFF16; IFTB, inoculated with *F. equiseti* VFF16 and treated with *B. amyloliquefaciens* VFS2; IFTF, inoculated with *F. equiseti* VFF16 and treated with fungicide; NINT, non‐incoculated and non‐treated; NITB, and non‐inoculated and treated with *B. amyloliquefaciens* VFS2. The experiment was performed in a greenhouse and plants were submitted to unstressed conditions, under salt stress (1.8 ‐ 1.9 ms m^−1^ in soil) and under drought stress (50% water holding capacity). Five replicates were considered for each treatment. The statistical differences under the same growth condition (Control, Drought stress, Salt stress) are indicated with letters of significance in black for the control condition; dark grey for PEG6000, and light grey for NaCl. The *P*‐values illustrated in the figure indicate the significance of differences for each treatment between the three incubation conditions (Tukey HSD test at *P* = 0.05). Treatments: IF, Inoculated with *F. equiseti* VFF16; IFTB, Inoculated with *F. equiseti* VFF16 and treated with *B. amyloliquefaciens* VFS2; IFTF, Inoculated with *F. equiseti* VFF16 and treated with fungicide Benomyl; NINT, non‐inoculated and non‐treated; NITB non‐inoculated and treated with *B. amyloliquefaciens* VFS2. *P*‐values indicate the significant difference in each treatment between the three incubation conditions (Control, salt and drought stress). Letters indicate the significant difference of the treatments in each incubation condition (Control, salt and drought stress) for Root and Shoot dry weight.

As a first comparison, the effect of stress condition of each treatment alone, a significant effect of stress condition on SDW for treatments IFTB (*P* < 0.01) and IF (*P* < 0.01) was noticed. Where for RDW, significant differences were noticed for the treatments IF (*P* < 0.01), IFTB (*P* < 0.01), IFTF (*P* < 0.01), and NINT (*P* < 0.01) (Fig. 5, Table S1). The results showed a significant decrease in RDW (*P* < 0.01) and SDW (*P* = 0.008) for the IF treatment, compared to the control treatment (*IF vs. NINT*) under the three systems. As a biocontrol essay, the RDW of IFTB was significantly higher than IF plants (*INTB vs. IF*) under unstressed (*P* < 0.001), salt (*P* < 0.001) and drought (*P* < 0.001) systems. Similarly, SDW for IFTB treatment was significantly higher than IF plants under unstressed (*P* < 0.001), salt (*P* < 0.001) and drought (*P* < 0.001) systems, confirming the efficacy of the strain VFS2 (Fig. 5). Besides, the inoculation of *B. amyloliquefaciens* VFS2 to *F. equiseti* VFF16 in pathogen‐inoculated plants showed no significant difference of RDW with control plants (*IFTB vs. NINT*) and the fungicide treatment (*IFTB vs. IFTF*) under unstressed (*P* > 0.05), salt (*P* > 0.05) and drought (*P* > 0.05) stress conditions. Likewise, SDW was not significantly different from control plants and the fungicide treatment (*IFTB vs. IFTF*) under unstressed (*P* > 0.05), salt (*P* > 0.05) and drought (*P* > 0.05) stress conditions.

The disease reduction percentage for the RDW in the IFTB treatment was about 79% under the unstressed conditions and increased to 100% under salt and drought stress conditions (Fig. 5, Table S1). For SDW, the disease reduction percentage (DR%) was about 71% for IFTB treatment under unstressed conditions (*vs*. 55% for the IFTF treatment) and reached a percentage of 100% under drought stress [*vs*. 81% for the fungicide treatment (IFTF)]. Also under salt stress, the DR% of SDW for the strain VFS2 treatment was higher (with 64%) than the fungicide treatment, which was equal to 57%, compared to non‐inoculated and non‐treated plants (NINT).

## 4 DISCUSSION

*Bacillus amyloliquefaciens* VFS2 was selected among a large collection of *Bacillus sp*. strains associated with *V. faba* plants, for its broad‐spectrum of antifungal secondary metabolites production and its growth inhibition capacity against phytopathogenic fungi affecting *V. faba*.^25^ The present study assessed the disease control and secondary metabolite production efficacy of *B. amyloliquefaciens* VFS2 strain under salt and drought stress. Previous reports have proposed *B*. *amyloliquefaciens* as a model bacterium species for plant growth promotion and to improve plant resistance to environmental stress.^37^, ^38^ The orientation of the present study to determine the effects of drought and salt stress on the fitness of *B. amyloliquefaciens* VFS2 is prompted by the current global climate change that could have an impact on plant‐pathogen‐biocontrol agent interactions. Recent investigations showed that plant diseases caused by various fungal pathogens may result in higher damage to crops under climate change conditions.^39^ Therefore, the development of some control measures for plant pathogens under extreme conditions is a necessary issue.

The *in vitro* growth of strain VFS2 is maintained in the presence of different concentrations of NaCl up to 200 mM and PEG6000 up to 20% at the same AUGC and AUSC values as those obtained for control non‐amended medium, highlighting its osmoadaptation potential under salt and drought conditions. The osmoadaptation of plant‐beneficial bacteria was reported as an important trait that determines their survival and effectiveness under field conditions.^14^ The *B. amyloliquefaciens* VFS2 exhibited higher antifungal activity against eight phytopathogenic fungi when grown at high salt concentrations (200 mM) and PEG6000 (20%). Interestingly, the strain VFS2 showed an increase in the production of cLPs as iturins, fengycins and surfactins on the growth medium amended with different concentrations of NaCl and PEG6000 in comparison to the non‐amended controls. The iturins, fengycins, and surfactins play a key role in the antifungal activity of *Bacillus* sp., and their synergetic effect was proved in recent reports.^5^, ^15^ Furthermore, it was reported that cLPs play a role in the survival of *Bacillus* strains in their habitat, increasing the bioavailability of hydrophobic water‐insoluble substrates, quorum sensing, motility, and biofilm formation involved in plant root colonization.^5^, ^40^

*Bacillus amyloliquefaciens* VFS2 showed an increase in IAA and siderophores production under salt and drought stress compared to non‐stressed conditions, known for their regulation of plant growth as well as of the antifungal activity.^41^, ^42^ The increased production of auxins and siderophores under abiotic stress may be an advantage for this strain to exhibit its PGP potential. It was recognized that PGP traits in *Bacillus* species promote plants to adapt to adverse abiotic conditions,^43^ which cannot be accomplished by agrochemicals.^5^ This beneficial effect has been confirmed in the present work in the biocontrol assay of *F. equiseti* VFF16 using the *B. amyloliquefaciens* VFS2 under salt and water deficiency regimes performed on *V. faba*. *Fusarium equiseti* VFF16 significantly reduced the SDW and RDW of *V. faba* under salt and drought stress, compared to the control, confirming the increased pathogenicity of this pathogen under abiotic stress conditions. However, the inoculation of VFS2 to *F. equiseti* VFF16‐infected plants re‐established the growth of the plant host to values obtained in non‐inoculated and non‐treated plants (control). The effectiveness of VFS2 can be attributed to its osmoadaptation ability to abiotic stress as well as to its capacity to produce a large spectrum of secondary metabolites involved in the accomplishment of its PGP potential. Several reports emphasize that the production of secondary metabolites with antifungal properties by *Bacillus sp*. in soil‐borne disease‐infested soils may confer this beneficial bacterium an advantage for promoting plant growth, which in turn could have a better ability to withstand salinity and drought compared with other PGPR.^13^, ^41^

## 5 CONCLUSION

The study demonstrated that *B. amyloliquefaciens* VFS2 exhibits osmoadaptation capacity under salt and drought stress. It maintained strong antifungal activity against various pathogenic fungi of *V. faba* plants and produced significant amounts of cLP isoforms (iturins, fengycins, surfactins), indole‐3‐acetic acid, and siderophores under osmotic stress. This capability enhanced the biocontrol of *Fusarium* wilt in *V. faba* under saline and water‐deficient conditions in the greenhouse experiment. Consequently, *B. amyloliquefaciens* VFS2 is a promising candidate for biocontrol of *Fusarium* wilt diseases amid the challenges of climate change. This finding led us to further develop strain VFS2 as a microbial biopesticide and plant growth promoter for field applications.

## AUTHOR CONTRIBUTIONS

Imen Hadoudi performed the main experiments and data analyses, and wrote the manuscript. Jordi Cabrefiga, Isabel Mora and Haythem Mhadhbi assisted in laboratory experiments and data analyses. Moncef Mrabet and Emilio Montesinos designed the research, obtained financial support and provided labs, contributing to the writing process.

## FUNDING INFORMATION

This work was supported by grants from the Ministry of Higher Education and Scientific Research of Tunisia (Grant LL2015‐2019) and a scholarship by University Tunis El Manar, Tunisia.

## CONFLICT OF INTEREST

The authors declare no conflict of interest.

## Supporting information


**Data S1.** Supporting Information.

**Data S2.** Supporting Information.

## Data Availability

The data that support the findings of this study are available from the corresponding author upon reasonable request.
