# Supplementary material for: Investigating Bacillus amyloliquefaciens VFS2 for Vicia faba‐fusarium wilt biocontrol and plant growth promotion under osmotic stress
Source: Pest Manag Sci. 2025 Jul 24;81(12):7665–74. doi: 10.1002/ps.70078 (PMC12618912; doi:10.1002/ps.70078)
Supplement: Supplementary file 1 — Data S1. Supporting Information. [file PS-81-7665-s002.docx]

**Investigating *Bacillus* *amyloliquefaciens* VFS2 for *Vicia* *faba*-*Fusarium* wilt biocontrol and plant growth promotion under osmotic stress**

**Supplementary Figures caption**

**Supplementary Fig. S1** Antagonistic activity of *B. amyloliquefaciens* VFS2 on PDA medium added NaCl (200 mM) for salt stress and PEG6000 (20%) for drought stress against *Rhizopus oryzae* VFF1 (a), *Rutstroemia* sp. VFF7 (b), *Boremia exigua* VFF4 (c), *F. equiseti* VFF16 (d), *F. graminearum* (e), *F. brachygibbosum* VFF2 (f), *F. oxysporum* KLR14 (g), and *F. equiseti* VFF12 (h).

**Supplementary Fig. S2** Root growth in the biocontrol assay of *F. equiseti* VFF16 in V. *faba* using *B. amyloliquefaciens* VFS2 under unstressed (a), salt-stress (b), and drought-stress (c) conditions. IF, inoculated with *F. equiseti* VFF16; IFTB, inoculated with pathogenic fungi and treated with *B. amyloliquefaciens* VFS2; IFTF, inoculated with *F. equiseti* VFF16 and treated with fungicide; NINT, non-inoculated and non-treated; NITB non-inoculated and treated with *B. amyloliquefaciens* VFS2.

| **Control**  **a** | **NaCl (200 mM)** | **PEG6000 (20%)** | **Control**  **e** | **NaCl (200 mM)** | **PEG6000 (20%)** |
| --- | --- | --- | --- | --- | --- |
| 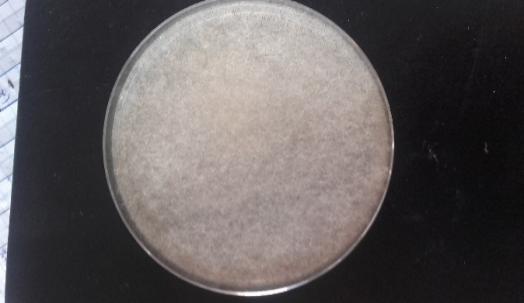  **b** | 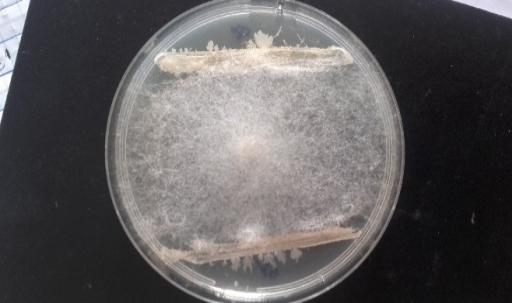 | 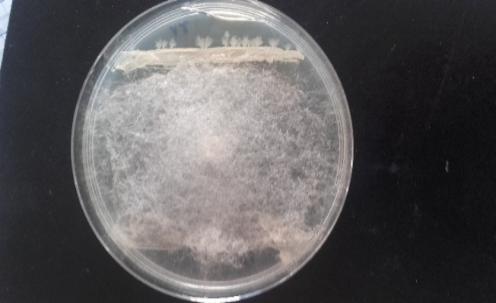 | 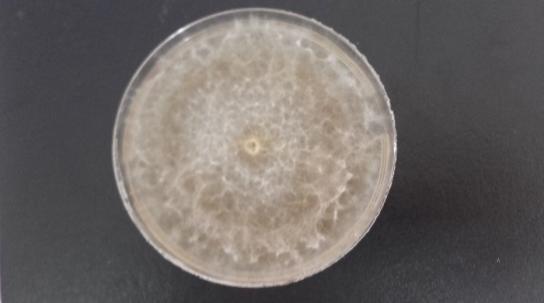 | 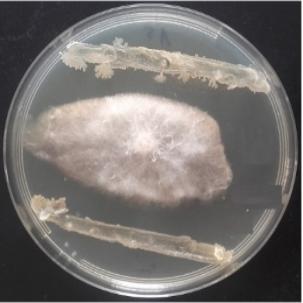 | 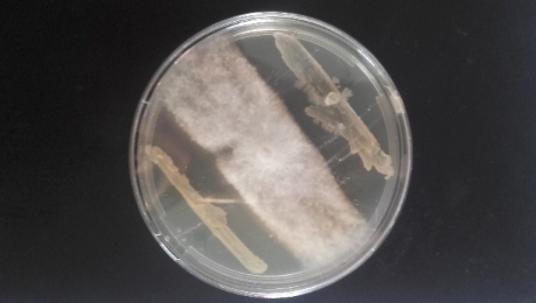 |
| 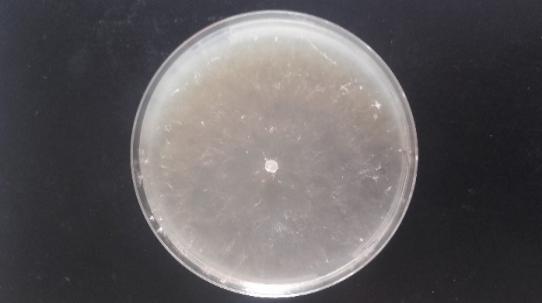 | 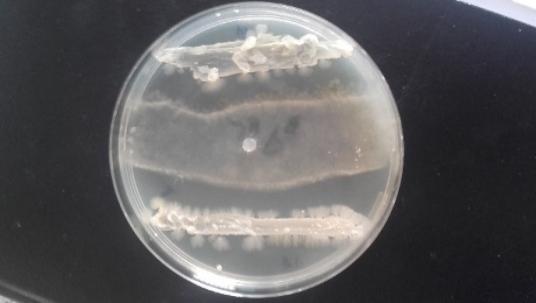 | 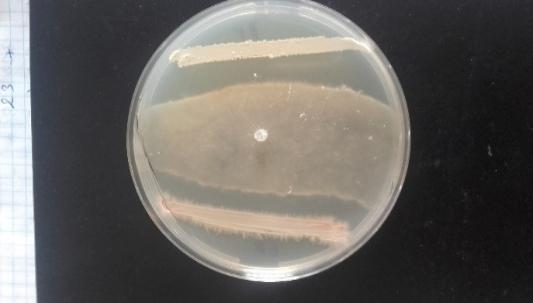 | 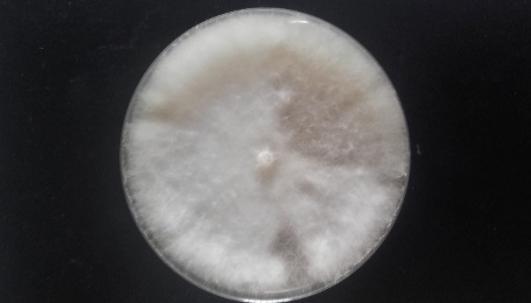  **f** | 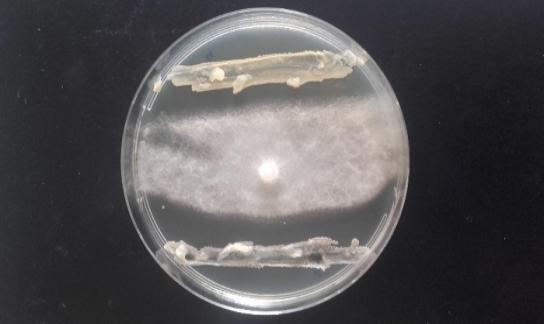 | 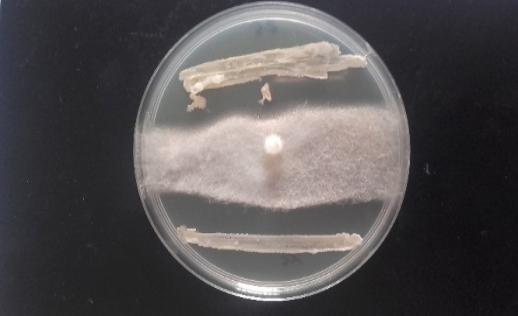 |
| 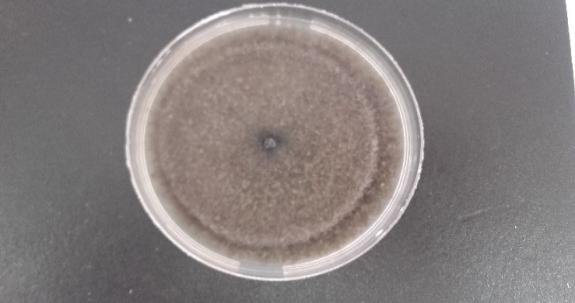  **d**  **c** | 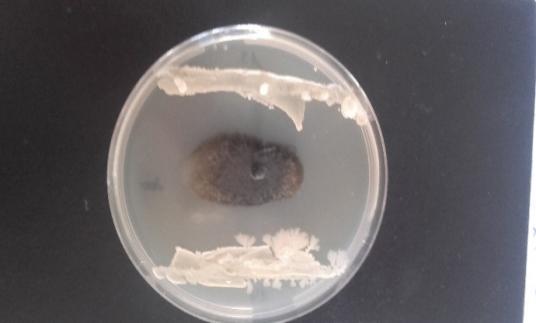 | 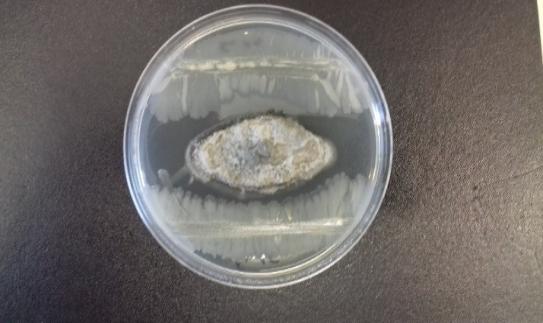 | 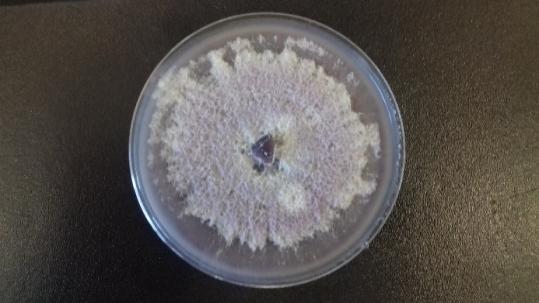  **h**  **g** | 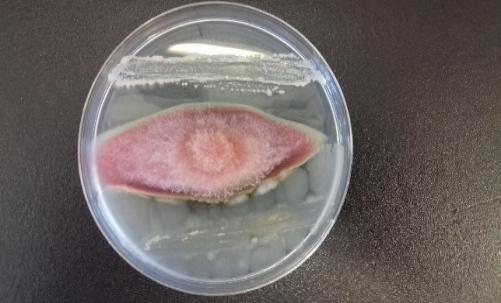 | 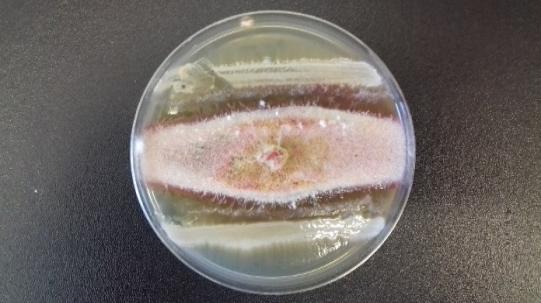 |
| 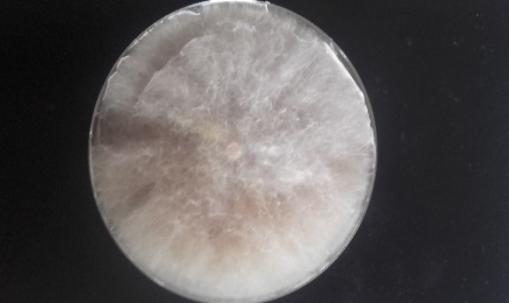 | 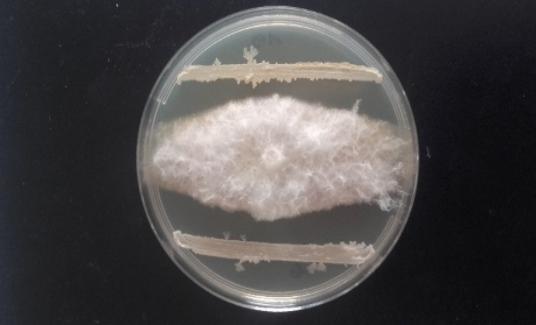 | 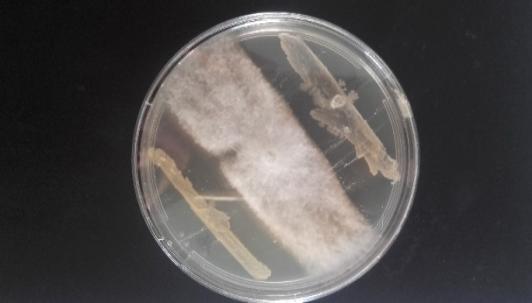 | 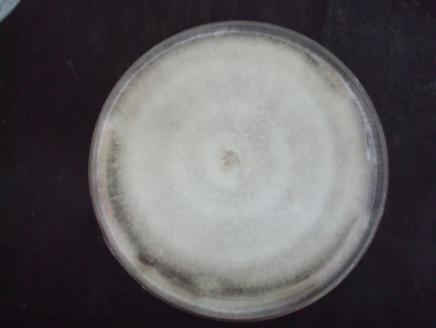 | 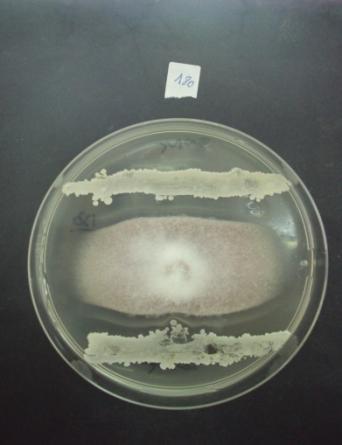 | 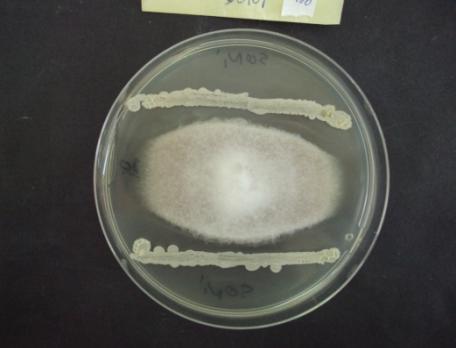 |

**Supplementary Fig. S1**


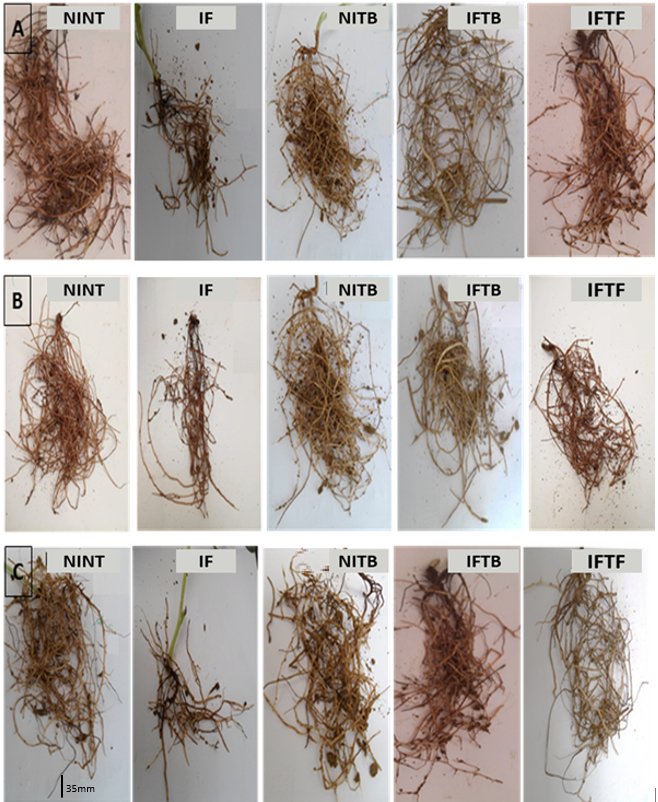


**Supplementary Fig. S2**
