# Supplementary material for: Investigating Bacillus amyloliquefaciens VFS2 for Vicia faba‐fusarium wilt biocontrol and plant growth promotion under osmotic stress
Source: Pest Manag Sci. 2025 Jul 24;81(12):7665–74. doi: 10.1002/ps.70078 (PMC12618912; doi:10.1002/ps.70078)
Supplement: Supplementary file 2 — Data S2. Supporting Information. [file PS-81-7665-s001.docx]

**Investigating *Bacillus* *amyloliquefaciens* VFS2 for *Vicia* *faba*-*Fusarium* wilt biocontrol and plant growth promotion under osmotic stress**

**Supplementary Table 1.** Faba bean root and shoot dry weight for the five treatments under the three growth conditions (control, salt and drought stress conditions).

| Incubation Condition | Treatment | Dry weight (mg/plant) | | |  | |
| --- | --- | --- | --- | --- | --- | --- |
| Root Shoot | | | | | |  |
| Control | IF | 968.5±24^a^ |  | 1378.5±54^a^ |  | |
|  | IFTB | 818.4±9.99^ab^ |  | 1279.0±67^a^ |  | |
|  | NITB | 664.6±61.4^abc^ |  | 1197.6±35^a^ |  | |
|  | NTIF | 615.6±21^bc^ |  | 1085.8±65^a^ |  | |
|  | NTNI | 363.3±31^c^ |  | 702.0±23^b^ |  | |
| Salt  (1.9 ms.m^-1^) | IF | 1028.9±45^a^ |  | 1243.0±56^a^ |  | |
|  | IFTB | 755.4±21^b^ |  | 1137.5±45.3^a^ |  | |
|  | NITB | 545.0±13^c^ |  | 1090.6±56.9^a^ |  | |
|  | NTIF | 504.5±15^c^ |  | 1082.7±23.9^a^ |  | |
|  | NTNI | 235.2±9.5^d^ |  | 660.1±10^b^ |  | |
| Drought (50%WHC) | IF | 866.7±10^a^ |  | 1446. ±34**^a^** |  | |
|  | IFTB | 667.6±8.9^b^ |  | 1333.6±54.5^a^ |  | |
|  | NITB | 666.7±11^b^ |  | 1303.1±43^a^ |  | |
|  | NTIF | 635.6±12^b^ |  | 843.0±12^b^ |  | |
|  | NTNI | 186.1±7.3^c^ |  | 464.5±12^c^ |  | |

Each value represents the mean of five replicates ±SD

Different letters showed significant differences among treatments at the same incubation condition according to the Tukey HSD test at P=0.05.

Treatment: NINT: non-inoculated and non-treated; IF: non-treated and inoculated with *F. equiseti*; NITB: Treated only with *B. amyloliquefaciens* VFS2; IFTB: inoculated with *F. equiseti* and treated with strain VFS2; IFTF: inoculated with *F. equiseti* and treated with the fungicide Benomyl (0.5 g.L^-1^)
